# Acute Frontal Lobe Dysfunction Following Prefrontal Low-Frequency Repetitive Transcranial Magnetic Stimulation in a Patient with Treatment-Resistant Depression

**DOI:** 10.3389/fpsyt.2017.00096

**Published:** 2017-05-30

**Authors:** Guilhem Carle, Mehdi Touat, Nicolas Bruno, Damien Galanaud, Charles-Siegfried Peretti, Antoni Valero-Cabré, Richard Levy, Carole Azuar

**Affiliations:** ^1^AP-HP, Hôpital Saint-Antoine, Department of Psychiatry, Paris, France; ^2^FrontLab, INSERM U1127, CNRS UMR7225, IHU Translational Neurosciences, Institut du Cerveau et de la Moelle Epinière (ICM), Paris, France; ^3^Sorbonne Universitas Pierre et Marie Curie (UPMC) University, Paris, France; ^4^AP-HP, Hôpital Saint-Antoine, Department of Neurology, Paris, France; ^5^Paris Sud University, Gustave Roussy, INSERM U981, Villejuif, France; ^6^AP-HP, Groupe Hospitalier Pitié-Salpêtrière, Department of Neuroradiology, Paris, France; ^7^Laboratory for Cerebral Dynamics Plasticity and Rehabilitation, School of Medicine, Boston University, Boston, MA, USA; ^8^Cognitive Neuroscience and Information Technology Research Program, Open University of Catalonia (UOC), Barcelona, Spain; ^9^AP-HP, Groupe Hospitalier Pitié-Salpêtrière, National Reference Centre on Rare Dementias, Paris, France

**Keywords:** depression, transcranial magnetic stimulation, frontal syndrome, antidepressant, state-dependency, executive dysfunction

## Abstract

The potential of repetitive transcranial magnetic stimulation (rTMS) to treat numerous neurological and psychiatric disorders has been thoroughly studied for the last two decades. Here, we report for the first time, the case of a 65-year-old woman suffering from treatment-resistant depression who developed an acute frontal lobe syndrome following eight sessions of low-frequency rTMS (LF-rTMS) to the right dorsolateral prefrontal cortex while also treated with sertraline and mianserin. The pathophysiological mechanisms underlying such an unexpected acute frontal lobe dysfunction are discussed in relation to the therapeutic use of LF-rTMS in combination with pharmacotherapy in depressed patients.

## Background

Over the past two decades, repetitive transcranial magnetic stimulation (rTMS) has shown promise in the treatment of a myriad of neurological and psychiatric conditions such as Parkinson’s disease, neuropathic pain, stroke, depression, and auditory hallucinations (1, 2). rTMS involves the use of a magnetic field to induce an electric current in cortical regions, in order to modulate their activity levels and those of their associated networks. Different stimulation parameters are combined in order to induce lasting increases or decreases of neuronal activity. rTMS was approved in 2008 by the Food and Drug Administration as a treatment for major depressive disorder (MDD) in patients who did not respond to at least one antidepressant medication (1–3). In a recent meta-analysis of MDD and treatment-resistant depression [TRD (4)], the administration of low-frequency rTMS (LF-rTMS) to the right dorsolateral prefrontal cortex (rDLPFC) proved efficacy in 30–40% of patients. Despite some rare adverse effects [including seizures, local pain, transient hypomanic or manic episode, and delusion (1, 5–8)], rTMS is then considered a well-tolerated method to treat depression. Herein, we report the first case of acute frontal lobe dysfunction induced by a concurrent administration of LF-rTMS and antidepressant treatment in a TRD patient. Neuropsychological and neuroimagery investigations revealed a temporal association between transient clinical symptoms and reversible brain alterations. We further discuss the pathophysiological hypothesis of such acute frontal lobe dysfunction, and the specificity of the delivery of rTMS concurrently with the administration of psychotropic medication. Our patient gave written consent to publish this case report.

## Case Presentation

### History and Symptoms at Presentation

A 65-year-old right-handed retired woman presented with a depressive relapse. Her medical history revealed hypertension, hyperlipidemia, and rheumatoid arthritis in clinical remission for 6 years (DAS 28 score: 1.74), treated weekly with methotrexate [15 mg once daily (od)]. She reported neither a history of addictive behavior nor any neurological medical condition. She suffered from a TRD of 25 years (recurrent resistant episodes, with resistance to several types of antidepressant treatment, including resistance to electroconvulsive therapy), without any event of suicidal behavior or hypomanic/manic episode. She was treated with fluoxetine (40 mg od) and clomipramine (75 mg od) before relapsing. At presentation, she reported sadness, loss of interest, psychomotor retardation, thoughts of worthlessness, hyporexia, and difficulty concentrating. No agitation, irritability, insomnia, confusional state, or suicidal thoughts were reported. She was diagnosed with a severe TRD according to the Diagnostic and Statistical Manual of Mental Disorders IV-TR.

### Clinical Course and Management

Considering her TRD and clomipramine-induced orthostatic hypotension, treatment was progressively switched to mianserin, sertraline, and diazepam. LF-rTMS was initiated 2 weeks after the onset of the new pharmacological treatment [at an intermediary dosage, mianserin (10 mg od), sertraline (50 mg od), and diazepam (2 mg od)], over the rDLPFC [Magstim Rapid^2^ stimulator (Whitland, UK, www.magstim.com), using a 70-mm diameter figure-of-eight coil, at 110% of resting motor threshold, with continuous patterns at 1 Hz (1,000 pulses/day) which followed international guidelines for clinical rTMS uses (1)].

The cortical target was defined as the scalp location 6 cm rostral and in a parasagittal plane from the hotspot of the abductor pollicis brevis muscle in the primary motor cortex (M1). Pretreatment assessments including standard blood tests, brain computerized tomography scanner, and electroencephalography (EEG) were normal.

After the eighth rTMS session, the patient presented significant behavioral changes, including disinhibition, loss of manners, decrease in personal grooming, perseverative behaviors, hyperorality with sweet cravings, and impulsive actions (the patient ran away from the hospital to buy large amounts of chocolate at the supermarket). Aspontaneity and economy of speech without aphasia were also observed. She did not exhibit elevated mood, tachypsychia, logorrhea, or insomnia, excluding a diagnosis of hypomanic/manic episode. Moreover, the patient did not report any delusion or hallucination. On a neurological evaluation, the patient was alert and oriented, without any motor or sensitive deficit. However, the patient exhibited signs of right frontal lobe dysfunction including distractibility, environmental dependency, and left-sided visuospatial neglect (9).

**Figure 1:**
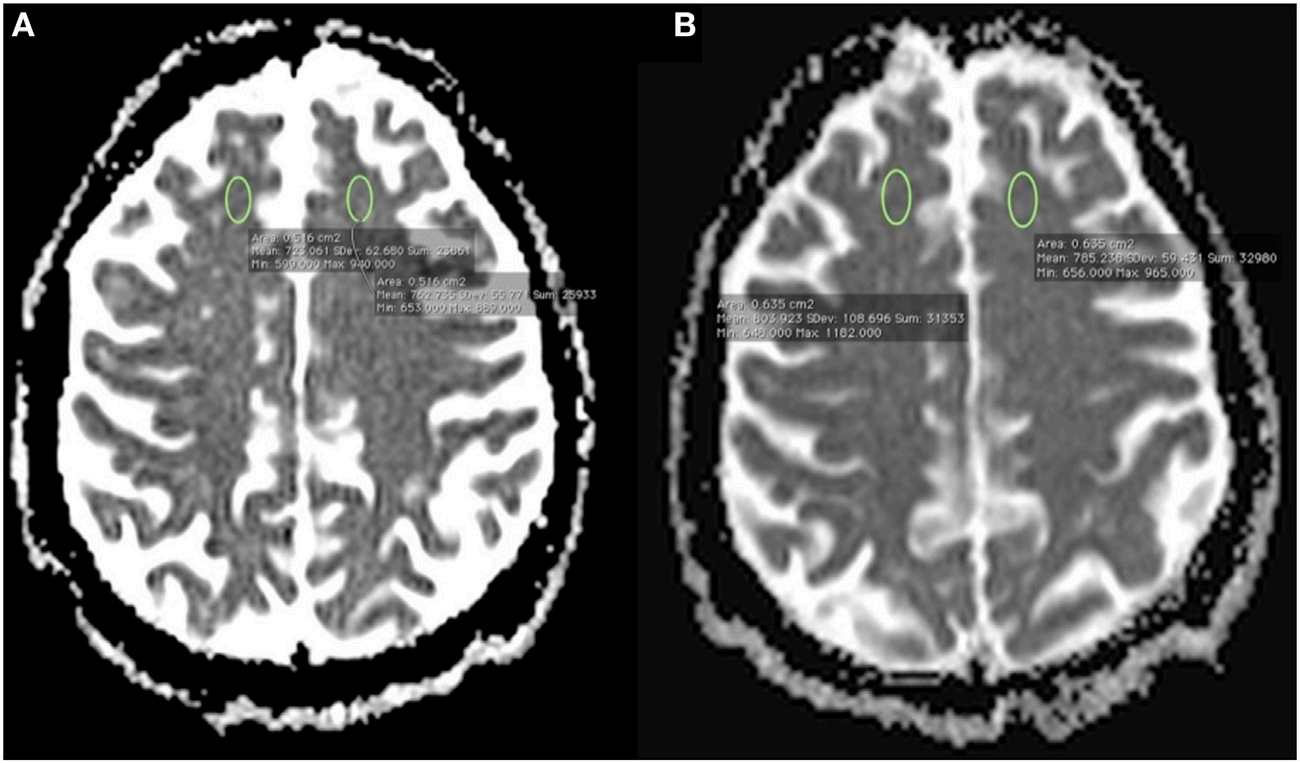
**(A)** 1.5 T brain magnetic resonance imaging (MRI) performed on day 1 showing a right dorsolateral prefrontal cortex (rDLPFC) hyperintensity on diffusion-weighted imaging with a significant decreased apparent diffusion coefficient (ADC) as compared to the homotopic region in the left hemisphere [difference of 0.039 × 10^−3^ mm^2^/s, showing 0.723 in the rDLPFC versus 0.762 in the left DLPFC (×10^−3^ mm^2^/s)]; **(B)** control brain MRI performed on day 9 after the onset of symptoms using the same MRI scanner and identical acquisition parameters revealing normal ADC levels on both DLPFC areas [no significant difference, showing 0.803 in the rDLPFC versus 0.785 in the left DLPFC (×10^−3^ mm^2^/s)].

Considering this acute frontal syndrome, we conducted an extensive checkup in order to rule out any acute neurological etiology. Epilepsy was ruled out with an EEG conducted during the acute symptomatic phase, revealing no ictal activity, notably in the frontal and temporal areas (even after provocation with hyperventilation test and flicker-fusion test). A second control EEG was conducted 7 days after the first EEG, revealing no abnormalities. Viral and bacterial encephalitis were ruled out by normal blood tests and cerebral spinal fluid (CSF) analysis [normal cell counts including no pleocytosis (0 cells), normal CSF protein level (0.33 g/L), normal CSF glucose level (3.5 mmol/L), negative HSV1/2-PCR, negative 14-3-3 protein]. General metabolic disturbances were ruled out by normal laboratory tests (normal glycemia, calcemia, and natremia). In order to rule out the hypothesis of an acute ischemic stroke, a 1.5 T brain magnetic resonance imaging (MRI) was performed on day 1. Right DLPFC hyperintensity on diffusion-weighted imaging was reported with a significant decreased apparent diffusion coefficient (ADC) as compared to the homotopic region [difference of 0.039 × 10^−3^ mm^2^/s, showing 0.723 in the rDLPFC versus 0.762 in the left DLPFC (×10^−3^ mm^2^/s), see Figure 1]. Nonetheless, these findings lacked an association with a high signal in FLAIR sequence more than 24 h after the onset of symptoms, and ADC level in our patient was higher than ADC level seen in patients suffering from an acute ischemic stroke (ADC mean in acute ischemic stroke = 0.533 × 10^−3^ mm^2^/s, SD = 0.157, 95% confidence intervals: 0.501–0.563) (10). The hypothesis of an acute ischemic stroke was then ruled out. In the context of rheumatoid arthritis, it was important to rule out the hypothesis of a cerebral vasculitis. First, considering rheumatoid arthritis, our patient had been in clinical remission for 6 years. There was no systemic sign of inflammatory activity, on a clinical and paraclinical level (including fever, fatigue, arthralgia, edema, cardiopulmonary function, renal and hepatic function, peripheral neuropathy, ophthalmic abnormalities). Second, blood tests were unremarkable {no inflammatory syndrome [normal C-reactive protein, normal white blood cell count, normal albuminemia (38 g/L)], normal serum protein electrophoresis}, and there was no inflammatory process revealed by CSF analysis (as already described). Furthermore, ADC level was not consistent with ADC level seen in acute ischemic stroke of any kind, including acute stroke induced by cerebral vasculitis (10). As the patient was treated with methotrexate, we aimed to address its imputability in the occurrence of such acute frontal syndrome. Methotrexate intake has always been well tolerated in our patient for 6 years. Our patient did not show any hypersensitivity reaction to methotrexate. She was supplemented with B vitamins, and laboratory tests revealed no vitamin deficiency. Finally, the period of time between the last intake of methotrexate and the acute phase had to be taken into account. Nonetheless, the reversibility of frontal symptoms was not correlated with a modification of this specific treatment. All put together, there was no argument in favor of a methotrexate-induced encephalitis.

**Figure 2:**
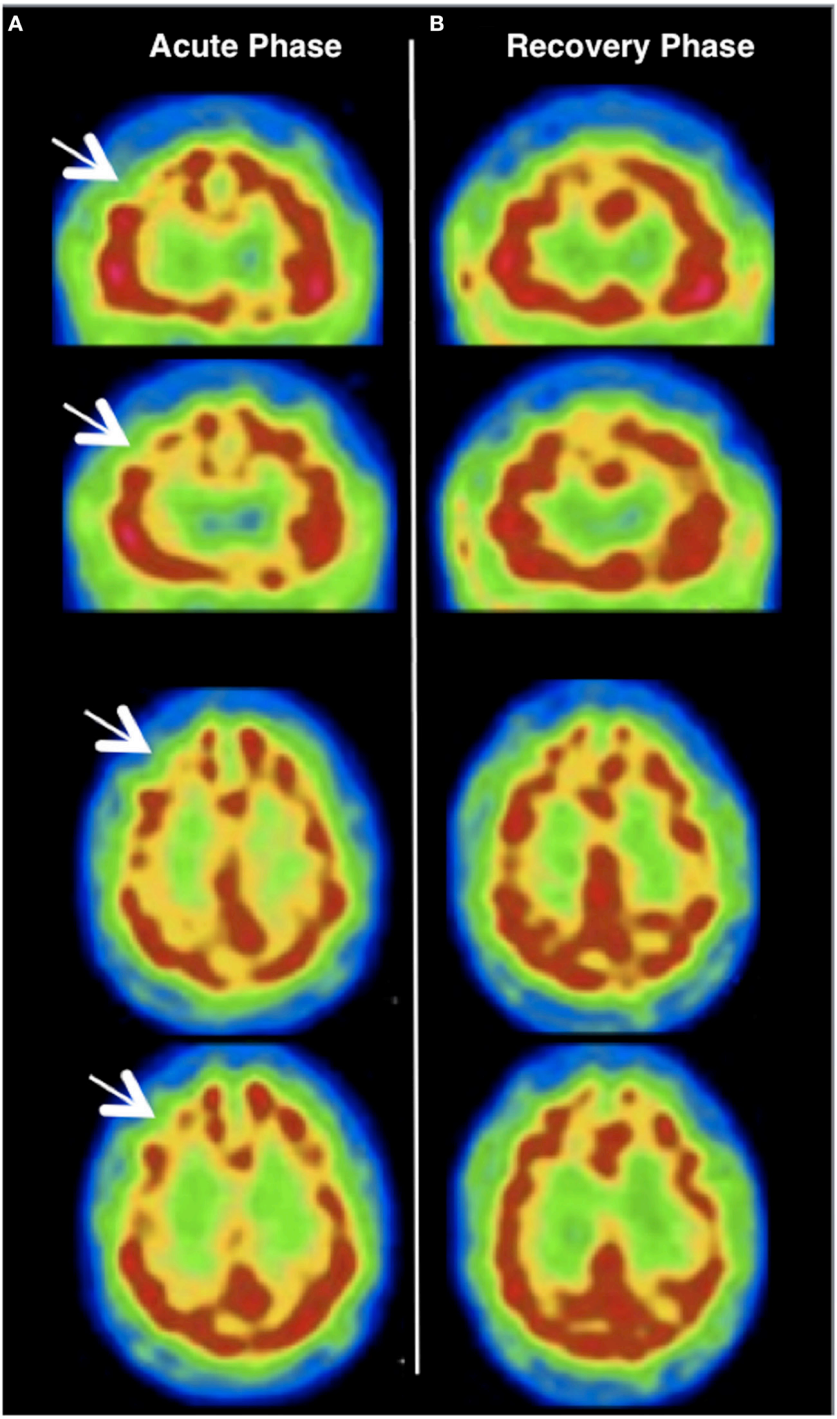
**(A)** Brain 99mTc-hexamethylpropyleneamine oxime single-photon emission computerized tomography (99mTc-HMPAO SPECT) performed 13 days after the onset of symptoms revealing a significant decrease in regional cerebral blood flow (rCBF) within the right dorsolateral prefrontal cortex (see white arrows); **(B)** a 10-month control 99mTc-HMPAO SPECT showing a complete cancelation of the right frontal rCBF decrease present during the symptoms.

After having ruled out these hypotheses, we tried to specify the frontal lobe dysfunction by conducting a neuropsychological evaluation in combination with other brain imaging techniques. Neuropsychological evaluation revealed deficits in executive and working memory tasks, without any instrumental impairment (see Table S1 in Supplementary Material). A brain 99mTc-hexamethylpropyleneamine oxime single-photon emission computerized tomography (99mTc-HMPAO SPECT) performed 13 days after the onset of symptoms revealed a significant decrease in regional cerebral blood flow (rCBF) within the rDLPFC (see Figure 2).

Repetitive TMS and sertraline were discontinued and the patient’s frontal lobe syndrome started to decline after 10 days off treatment. A control MRI performed on day 9 showed normal ADC levels in the rDLPFC [no significant difference, showing 0.803 in the rDLPFC versus 0.785 in the left DLPFC (×10^−3^ mm^2^/s), see Figure 1]. At the 3-month follow-up examination, the patient was stabilized on a regimen of mianserin 30 mg od in a complete recovery state, with a regression of the frontal dysfunction and a normalization of all neuropsychological functions. A 10-month control 99mTc-HMPAO SPECT revealed a complete cancelation of the rDLPFC rCBF decrease (see Figure 2).

## Discussion

Safety and potential side effects of LF-rTMS have been monitored clinically in thousands of depression patients and healthy controls to date (1, 2). Occasionally, studies have reported mild cognitive changes associated with the use of rTMS (improvement in number processing, memory recall, and creativity, or excessive tiredness, difficulty concentrating, and memory impairment), but these rare effects were subtle and lasted no more than several minutes (11–13). The current case report highlights the potential induction of an acute frontal lobe syndrome lasting up to a week, on a regime of LF-rTMS/antidepressant association.

We first placed particular emphasis on differential diagnosis. Psychiatric evaluation ruled out hypomania and mania, as described above. Moreover, the incidence of treatment-emergent mania is very low with rTMS and remains controversial (7). Nevertheless, we cannot exclude similar rTMS-induced cognitive dysfunctions as the one observed in our patient might have passed unnoticed. Indeed, some of the side effects of rTMS, such as agitation, hypomania, or mixed depressive episode, share core symptoms with a frontal lobe syndrome, and thus could easily be misdiagnosed (7, 8, 14, 15). Interestingly, all neurologic symptoms observed resolved shortly after the discontinuation of LF-rTMS and sertraline, without the use of specific therapeutics such as mood stabilizers.

Given the previous antidepressant switch in our patient, the risk for an antidepressant discontinuation syndrome (ADS) also needs to be considered. However, considering the 2-week latency between the antidepressant switch and the occurrence of symptoms, this possibility seems unlikely. Moreover, transient frontal lobe syndrome has never been reported in the context of an ADS (16).

Finally, as described above, behavioral changes, dysexecutive syndrome, and left-sided visual neglect exhibited by the patient strongly suggest a focal dysfunction associated with rDLPFC (9, 17). Remarkably, brain MRI and 99mTc-HMPAO SPECT revealed, respectively, transient diffusion and rDLPFC rCBF abnormalities overlapping with the area of LF-rTMS stimulation. These results raise the question of the correlation between clinical and abnormal imaging findings reported above. Previous TMS studies have also found a decrease in rDLPFC rCBF after LF-rTMS (18), and even a correlation between rDLPFC rCBF decreases and the efficacy of LF-rTMS (19). In addition, some studies outlined how LF-rTMS has the ability to modulate rCBF and brain diffusion and that such effects might scale with the clinical outcome (20, 21).

Considering the clinical presentation, what remains unclear here is the mechanism linking the LF-TMS-antidepressant treatment association to such acute behavioral degradation. SSRIs are preferentially known to induce indifference and apathy (22). Though, medication might have interfered with rTMS treatment. Mianserin and sertraline, alone or in combination, form a relative hazard for application of rTMS due to their significant seizure threshold lowering potential (1). Furthermore, recent research has demonstrated the strong state-dependency of the rTMS effects (23). The magnitude and even the net sign of the stimulation may depend on the state of activity of the targeted area. Thus, a brain region kept in a high state of activity may be more responsive to rTMS-mediated suppression than one with either normal or suppressed levels of activity (23). We therefore suggest that rTMS inhibitory effect might have been strongly potentiated through pharmacological preconditioning in our patient, lowering the excitability threshold of the rDLPFC and its associated network, hence inducing a transient change in the functioning of this neuronal network. Such widespread effect induced from a focal stimulation has been well described in animals and humans with positron emission tomography studies (24).

## Concluding Remarks

Repetitive transcranial magnetic stimulation/antidepressant treat-ment association is a very promising approach in the treatment of TRD. However, little is known on the neurophysiological mechanisms involved in such potentiation. To our knowledge, this is the first report of an acute frontal dysexecutive syndrome potentially mediated by rTMS/antidepressant treatment association in a TRD patient. Nonetheless, this report has to be considered with caution as this phenomenon occurred in a single case. Further rTMS/antidepressant studies are needed to explore such interaction and the mechanisms encountered in our patient. Moreover, our clinical observation provides support to adequately titrate rTMS/antidepressant treatment associations to boost the effects of rTMS for the therapy of TRD.

## Author Contributions

GC and MT contributed in drafting and revising the manuscript. All authors contributed in revising the manuscript and collecting the clinical data.

## Conflict of Interest Statement

The authors declare that the research was conducted in the absence of any commercial or financial relationships that could be construed as a potential conflict of interest.
