# Supplementary material for: Acute Frontal Lobe Dysfunction Following Prefrontal Low-Frequency Repetitive Transcranial Magnetic Stimulation in a Patient with Treatment-Resistant Depression
Source: Front Psychiatry. 2017 May 30;8:96. doi: 10.3389/fpsyt.2017.00096 (PMC5447704; doi:10.3389/fpsyt.2017.00096)
Supplement: Supplementary file 1 [file Table_1.DOCX]

| *Cognitive Function* | *Tests* | *Subtests* | *Patient's Score* | | |
| --- | --- | --- | --- | --- | --- |
|  |  |  |  |  |  |
|  |  |  | *Acute Phase* |  | *Recovery Phase* |
| ***Memory Tests*** |  |  |  |  |  |
| Verbal Episodic Memory | FCSRT-IR | Immediate Cued Recall | **11/16** |  | 14/16 |
|  |  | Identification | 16/16 |  | 16/16 |
|  |  | Total Free recall | **18/48** |  | 29/48 |
|  |  | Total (Free + Cued) Recall | **43/48** |  | 48/48 |
|  |  | Delayed Free Recall | **7/16** |  | 12/16 |
|  |  | Total Delayed Recall | 15/16 |  | 16/16 |
|  |  | Recognition | 16/16 |  | 16/16 |
| Autobiographic Memory |  |  | 5/5 |  | 5/5 |
| Semantic Memory |  |  | 4/5 |  | 5/5 |
| ***Memory Span Tests*** |  |  |  |  |  |
| Phonological Storage | Digit-Span |  | 7 |  | 8 |
| Central Executive | Backward Digit-Span |  | 4 |  | 5 |
| ***Executive Functions*** |  |  |  |  |  |
|  | FAB | Total | **14/18** |  | 18/18 |
| Conceptualization |  | Similarities | 3 |  | 3 |
| Mental Flexibility |  | Lexical Fluency | **2** |  | 3 |
| Environmental Autonomy |  | Prehension Behavior | 3 |  | 3 |
| Programming |  | Motor Series "Luria" Test | **1** |  | 3 |
| Sensitivity To Interference |  | Conflicting Instructions | 3 |  | 3 |
| Inhibitory Control |  | Go-No Go | **2** |  | 3 |
| Visuo-spatial Time Processing | TMTA | Time Performance/Numbers of Errors | **125''/3** |  | 53''/0 |
| Cognitive Set-Shifting | TMTB | Time Performance/Numbers of Errors | **>240''/7** |  | 73''/0 |
| Graphomotor processing speed | WAIS-III | Code | **33/133** |  | 47/133 |
| Sensitivity To Interference | Stroop | Reading | 28'' |  | NONE |
|  |  | Naming | 46'' |  | NONE |
|  |  | Interference | 72'' |  | NONE |
|  |  | Number of Unadjusted Errors | **7** |  | NONE |
| Mental Flexibility | Lexical Fluency | "M" Letter | **10** |  | 14 |
| Lexical Semantic Access | Categorial Fluency | "Animals" | 17 |  | 17 |
| Visuoconstructive skills | Rey Complex Figure | Copy | Type V; **18/36** |  | Type II; 30/36 |
| ***Instrumental Functions*** |  |  |  |  |  |
|  | Ideomotor Praxia |  | **10/12** |  | 12/12 |
|  | Semantic Praxia |  | 11/12 |  | 12/12 |
| Word finding difficulty/Paraphasia | Picture Naming |  | 12/12 |  | 12/12 |
|  | Semantic Matching |  | 12/12 |  | 12/12 |
|  | Acalculia | Calculation: 78-43 | normal |  | normal |

**Table 1 (supplementary material).** Characteristics and Neuropsychological Data of our patient in the Acute Phase and in the Recovery Phase.
